# Notifications registered by the National Hospital Epidemiological Surveillance Network (RENAVEH) during the COVID-19 pandemic

**DOI:** 10.1590/S2237-96222022000200026

**Published:** 2022-09-05

**Authors:** Thais Cláudia Roma de Oliveira Konstantyner, Suely Miyuki Yashiro, Nívia Aparecida Pissaia Sanches, Carla Gianna Luppi

**Affiliations:** 1 Universidade Federal de São Paulo, Departamento de Medicina Preventiva, São Paulo, SP, Brazil

Dear Editors,

Regarding the research note by Sallas et al.,^1^ we would like to make some comments aiming to shed light on epidemiological surveillance in the hospital context and enrich the debate on the theme.

The article corroborates the importance of Hospital Epidemiology Hubs (NHEs as per the Brazilian acronym) in the continuous monitoring of the epidemiological situation in the country, and its strategic role when facing public health emergencies, such as the current health scenario. In order for this to continue to occur in a timely and systematic manner, it is necessary to maintain and establish political-organizational strengths, as it can be seen in the implementation of the National Health Surveillance Policy (PNVS as per the Brazilian acronym),^2^ the National Network for Surveillance, Alert and Response to Public Health Emergencies of the Brazilian National Health System (VIGIAR-SUS as per the Brazilian acronym),^3^ the Hospital Epidemiological Surveillance (VEH as per Brazilian acronym)^4^ and the National Hospital Epidemiological Surveillance Network (RENAVEH as per the Brazilian acronym).^5^

It is worth mentioning that the recent reorganization of VEH associated the NHEs, linked to the Ministry of Health, to RENAVEH, and the main objective of which is to improve the capacity to detect, monitor and provide an immediate response to potential public health emergencies within hospital context and, thus, a great effort has been made to strengthen the network.

Sallas et al.^1^ concluded that the NHEs accounted on average for 8% of overall notifications recorded in the country and that there was a decrease in notifications of Diseases, Health Conditions and Public Health Events (DAEs as per the Brazilian acronym) in 2020. Although the information provided in the text is not clear, it allows us to infer that the number of notifications registered by the NHEs (n = 225,081) in 2020 did not include the cases of COVID-19 identified in the respective services, given that Brazil reported more than 7.6 million cases of the disease that year. If we consider this as the real context of analysis, it would be prudent to inform that, for the analysis of the pandemic period, only the DAEs that were on the national compulsory notification list of diseases in the pre-pandemic period were taken into consideration, excluding cases of COVID-19 (flu-like syndrome and severe acute respiratory syndrome). An unsuspecting reader or, in an awkward situation, a manager with limited resources could consider that there was a decrease in the workload of NHEs after the beginning of the pandemic, which is not consistent with the reality experienced by the vast majority of services in the country.

Finally, it is worth emphasizing the importance of periodic dissemination resulting from activities performed by the NHEs in the national territory. As such, we congratulate all authors and *Epidemiology and Health Services: journal of the Brazilian National Health System* (RESS) for the publication of the work. 
